# A Comparison of the Toxicities in Patients With Locally Advanced Head and Neck Cancers Treated With Concomitant Boost Radiotherapy Versus Conventional Chemoradiation

**DOI:** 10.7759/cureus.38362

**Published:** 2023-04-30

**Authors:** Ritu Sharma, Siddharth Vats, Rajeev Seam, Manish Gupta, Ratti R Negi, Vikas Fotedar, Kaalindi Singh

**Affiliations:** 1 Department of Radiotherapy, Shri Lal Bahadur Shastri Government Medical College and Hospital, Mandi, IND; 2 Department of Radiotherapy, Indira Gandhi Medical College, Shimla, IND; 3 Department of Radiotherapy, Maharishi Markandeshwar Institiute of Medical Sciences and Research, Ambala, IND

**Keywords:** quality of life, acute toxicity, locally advanced head and neck cancer, concomitant boost radiotherapy, concurrent chemo-radiation

## Abstract

Purpose: To compare the objective and patient-reported toxicities of concomitant boost radiotherapy (CBRT) and concurrent chemoradiation (CRT) in patients with locally advanced head and neck cancers.

Methods and material: In this prospective study, 46 patients with histologically proven stage III-IVA head and neck cancer were randomly assigned to receive either concurrent chemoradiation to a dose of 66 Gy in 33 fractions over 6.5 weeks with concurrent cisplatin (40 mg/m2 IV weekly; control arm) or accelerated radiotherapy with concomitant boost radiotherapy (study arm) to a dose of 67.5 Gy in 40 fractions in five weeks. Acute toxicity was evaluated using RTOG toxicity criteria. The assessment was done weekly after initiation of treatment, at the first follow-up (six weeks), and at three months. The four main patient-reported symptoms of pain, hoarseness of voice, dryness of mouth, and loss of taste were also compared between the two groups to assess patient quality of life during treatment.

Results: The mean treatment duration was 37 days in the CBRT arm and 49 days in the CRT arm. Treatment-related interruptions were less in the study group,17.3% in the study, and 27.2% in the control with insignificant P-value. Grade III laryngeal toxicity was significantly higher in the study group (P=0.029). Other acute grade I-III toxicities (pharyngeal, skin, mucositis, and salivary) were comparable in both CRT and CBRT arms. Grade IV toxicities were seen only in the CBRT arm but were resolved at the first follow-up. Haematological toxicities and renal toxicities were significantly higher in the CRT arm, with significant P-values of 0.0004 and 0.018, respectively.

Conclusion: In patients with locally advanced head and neck cancer, concomitant boost radiotherapy is well tolerated with acceptable local toxicity and minimal systemic toxicity as compared to conventional chemoradiation. It is a feasible option for patients with locally advanced head and neck cancer not fit for concurrent chemoradiation.

## Introduction

Head and neck malignancies constitute 6% of all cancers worldwide. As per the GLOBOCAN 2020 India factsheet, cancer of the lip and oral cavity is the 2nd (10.3%), the larynx the 11th (2.6%), the hypopharynx the 15th (2.2%), and the oropharynx the 18th (1.60%) most common cancer in Indian population [1]. At the Tertiary Care Cancer Centre, where this study was undertaken, head and neck cancers represented 10-15% of all cancers registered from 2010 to 2017.

The management of head and neck cancers requires a multidisciplinary approach. Surgery and radiotherapy (with or without chemotherapy) are the mainstays of treatment. Concurrent chemoradiation (CRT) is the standard organ preservation approach for patients with locally advanced head and neck cancer, as surgery is associated with significant morbidity. Multiple randomized trials and meta-analyses have shown that the addition of concurrent chemotherapy to radiotherapy improves survival and reduces locoregional recurrence, albeit with greater toxicity. An absolute benefit of 6.5% at five years has been shown in a meta-analysis of concurrent chemotherapy trials [2-7].

Altered fractionation schedules like hyperfractionation and accelerated fractionation have also been shown to improve the therapeutic ratio over conventional fractionation by either increasing the overall dose or overcoming accelerated repopulation. MARCH meta-analysis showed an absolute benefit of 3.4% at five years in the altered fractionation arm over conventional radiotherapy [8-24].

Among the altered fractionation regimens, accelerated fractionation with concomitant boost radiotherapy (CBRT) has the added advantage of early treatment completion, thus reducing patient waiting lists in busy departments. Several studies have shown modest improvement in loco-regional control with concomitant boost radiotherapy over conventional radiotherapy but with higher acute and late toxicity [12,25-27].

However, as concurrent chemoradiation is the standard of care in locally advanced head and neck cancer, this study was undertaken to compare the acute toxicities of CBRT and CRT and establish CBRT as a safe and feasible alternative to CRT in locally advanced head and neck cancer patients.

## Materials and methods

This trial was conducted in the Department of Radiotherapy at a tertiary care cancer centre. Appropriate Ethical Committee approval was obtained from the Institutional Review Board. Written informed consent was obtained from all participants.

**Figure 1:**
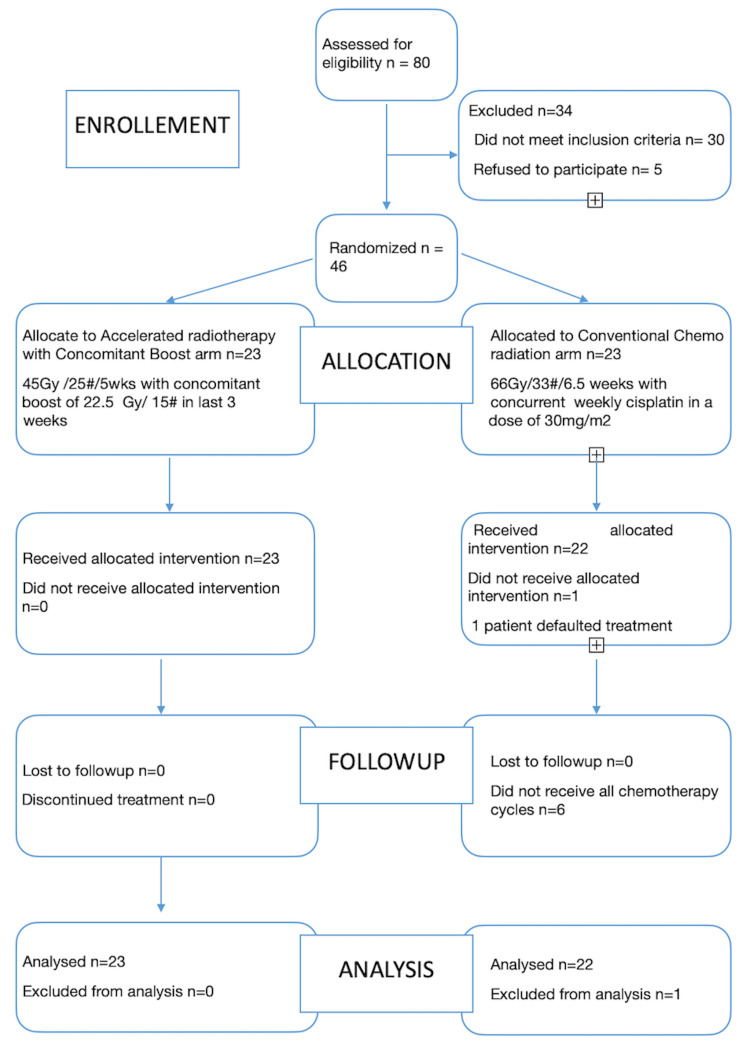
CONSORT flowchart of the study.

Enrollment was done for a period of one year, from July 2018 to July 2019. Patients with age ≤ 70 years, biopsy-proven stages III, IV A, IV B, oropharynx, hypopharynx, laryngeal squamous cell carcinomas, and Karnofsky performance status (KPS) >70 were included in the study. Exclusion criteria were - histology other than squamous cell carcinoma, impaired renal function and liver function, cytopenias, and previous treatment for malignancy or distant metastasis (Figure 1).

Randomization

Randomization was carried out by stratified randomization technique. Patients were randomized into study and control group in a 1:1 ratio. Equal numbers of patients were assigned to each group.

Study design

Control Arm (CRT Arm)

Patients in this arm were treated with standard chemoradiation at 66 Gy/6.5 weeks/33#. The radiation dose per fraction was 2 Gy with five fractions per week from Monday to Friday. Cisplatin at a dose of 30 mg/m^2^ was given weekly after routine blood investigations. Mannitol-containing formulations were preferred, otherwise, injection of Mannitol was given after Cisplatin infusion.

Study Arm (CBRT Arm)

**Figure 2:**
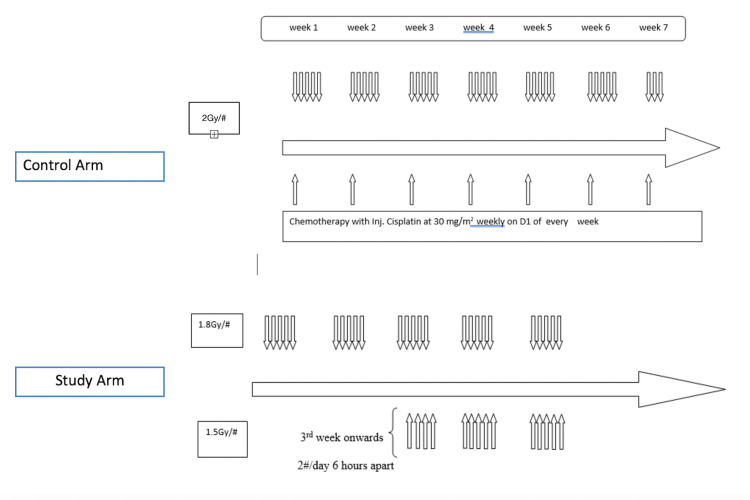
Schematic representation of treatment delivery in control and study arm.

Patients in this arm were treated with radiation alone at 67.5 Gy/40#/5 weeks in two phases. In phase I, 45 Gy/25#/5 weeks was given to the primary tumour and draining lymph nodes. In phase II, after 10 fractions, a concomitant boost of 22.5 Gy/15#/3 weeks was given to the gross disease and involved nodes with a margin of 1.5-2 cm (Figure 2).

Administration of radiotherapy treatment

Prior to radiotherapy initiation, dental clearance was obtained for all patients. Patients were advised not to shave or apply the cream before radiotherapy treatment. Immobilization was achieved by using a thermoplastic mask. Patients were given external beam radiotherapy by Theratron 780 E and Equinox Cobalt-60 machines using two parallel opposed fields by the "shrinking field" technique. The cord off was done after 45 Gy.

The biologically equivalent dose was calculated using a linear quadratic model with α/β value of 10 for tumour control probability in squamous cell cancers of the head and neck region and 3 for late-reacting normal tissues. The biologically effective dose (BED) for the tumour in the CRT arm was 79.2 Gy and 78.98 Gy in the CBRT arm while BED for late-reacting tissue was 110 Gy in the CRT arm and 105.75 Gy in the CBRT arm.

Supportive treatment

Topical gentian violet and topical antibiotics were given to treat radiation dermatitis when indicated. To prevent mucositis, patients were advised to maintain oral hygiene through frequent oral rinsing and gargling with diluted benzydamine solution. Topical anaesthetics, antibiotics, and antifungals were given as and when required. Odynophagia due to pharyngeal toxicity was treated with topical analgesics and narcotic and non-narcotic analgesics as per the WHO step ladder.

To maintain the nutrition and hydration of patients, oral and fluid intake was encouraged by providing diet sheets prepared by dieticians, and intake was monitored by diet charting. Oral supplements and intravenous fluids were given as and when required. A nasogastric feeding tube was inserted for grades III and IV pharyngeal toxicity. Laryngeal toxicities were carefully monitored and treated with antitussives, analgesics, steroids, and antibiotics if indicated. For dryness of the mouth, frequent oral sips were advised.

Toxicity assessment

Acute toxicities were assessed according to RTOG criteria (Radiation Therapy Oncology Group) [28]. Toxicities were documented weekly during treatment, at the first follow-up, i.e., at six weeks and at three months. Scoring was done according to the patient’s symptoms, examination findings, and treatment of symptoms.

Follow-up

The first follow-up was done six weeks after the completion of treatment. On follow-up, a complete history and physical examination were done, which included an oral and neck examination and indirect laryngoscopy. Patients were advised to have an X-ray chest or CT scan if clinically indicated. Toxicity that occurred within 90 days of treatment was considered acute and toxicities that lasted or occurred after 90 days were considered late toxicities.

Statistical analysis

The scores of acute radiation reactions in both arms were documented and compared. The data analysis was done on IBM SPSS statistics software version 26 (IBM Corp., Armonk, NY), using the chi-square test and t-test for qualitative and quantitative data, respectively. A p-value of <0.05 was taken as statistically significant.

## Results

Over 80 patients were assessed for eligibility, and 45 patients were found eligible as per inclusion and exclusion criteria. Of the 45 patients, 23 were randomized to the study arm (CBRT), and 23 were randomized to the control arm (CRT).

Most of the patients presented in the age group of 61-70 years. The median age of the presentation was 60 years. Both arms were well balanced with respect to age, gender, patient habits, oro-dental hygiene, site-wise distribution, and T and N stage of the disease. The most common site was the larynx (commonest subsite-supraglottis).

Response to treatment

At the first follow-up, i.e., six weeks, the response was assessed clinically. The overall response rate was 91.2% in the study arm and 95.3% in the control arm, p-value of 0.468. Regarding nodal disease, the overall response rate was 79.1% in the study arm and 72.6% in the control arm, p-value of 0.218. Thus, the response was comparable between the two arms at the first follow-up.

Toxicities during treatment

The highest toxicities documented during treatment and at follow-up were compared between the study and control arms. Grade III laryngeal toxicity was significantly higher in the study arm (P=0.029). Grades II and III pharyngeal and skin toxicities were higher in the study arm as compared to the control arm but the difference was statistically not significant. Grade IV skin toxicity was seen only in the study arm with an insignificant P-value. Grade III mucosal toxicities were more common in the study arm (P=0.211) and grade IV toxicity was seen in 1 patient in the study arm (P=0.645). Grade II salivary toxicity was higher in the study arm, however, the difference was statistically insignificant (Table 1).

**Table 1:** Summary of laryngeal, pharyngeal, skin, mucosal, and salivary toxicity during treatment.

Grade of toxicity	Study (%)	Control (%)	Total (%)	P-value
Laryngeal				
Grade I	9(39.1)	12(54.5)	21(46.6)	0.641
Grade II	9(39.1)	10(45.4)	19(42.2)	0.432
Grade III	5(21.7)	0	5(11.1)	0.029
Grade IV	0	0	0	NA
Pharyngeal				
Grade I	2(8.6)	8(36.3)	10(22.2)	0.364
Grade II	12(52.1)	10(45.4)	22(48.8)	0.671
Grade III	9(39.1)	5(22.7)	11(24.4)	0.473
Grade IV	0	0	0	NA
Skin				
Grade I	4(17.3)	9(40.9)	13(28.8)	0.535
Grade II	12(52.1)	10(45.4)	22(48.8)	0.170
Grade III	5(21.7)	3(13.6)	8(17.7)	0.612
Grade IV	2(8.6)	0	2(4.4)	0.560
Mucosal toxicity				
Grade I	2(8.6)	4(18.1)	6(13.3)	0.306
Grade II	11(47.8)	14(60.8)	25(55.5)	0.197
Grade III	9(39.1)	4(18.1)	13(28.8)	0.211
Grade IV	1(4.3)	0	1(2.22)	0.645
Salivary toxicity				
Grade I	7(30.4)	12(54.5)	19(42.2)	0.112
Grade II	16(69.5)	10(45.4)	26(57.7)	
Grade III and IV	0	0	0	NA

Haematological toxicity was significantly higher in the control arm, as chemotherapy was administered only in the control arm (P=0.0004). Nephrotoxicity was observed only in the control arm. Six patients had impaired renal functions, due to which seven complete cycles of chemotherapy could not be given. The difference was statistically significant (P-value=0.018) (Table 2).

**Table 2:** Haematological toxicity and radiotherapy interruption in both arms.

Toxicity	Study N(%)	Control N(%)	Total N(%)	P-value
Haematological				
Grade 0	18(78.2)	4(18.1)	22(48.8)	0.0004
Grade I	4(17.3)	13(59)	17(37.7)	
Grade II	1(4.3)	3(13.6)	4(8.88)	
Grade III	0	2(9)	2(4.4)	
Grade IV	0	0	0	NA
Radiotherapy interruption				
Yes	8(34.7)	4(18.1)	12(26.6%)	0.598
No	15(65.2)	18(81.8)	33(73.3)	

Radiotherapy was interrupted due to machine breakdown in 3(13%) patients in the study arm and 1(4.5%) patient in the control arm. Radiotherapy was interrupted due to treatment-related toxicities in 4(17.4%) patients in the study arm and 6(27.3%) patients in the control, one patient in the study arm defaulted treatment. The difference in the two arms was not significant (P-value=0.635).

Assessment of patient reported symptoms

The four most commonly reported symptoms of head and neck cancer patients during treatment were compared using Likert scale-based questions. Patients were asked about the intensity of throat pain, dry mouth, post-treatment hoarseness of voice, and loss of taste and patients had to choose a response from the following options - not at all, a little, quite a bit, and very much. The assessment was done weekly during the course of treatment, at six weeks and three months. The highest response was chosen for evaluation.

Pain throat assessment was comparable in both groups, but patients experiencing very much pain were higher (8.6%) in the study group as compared to the control group (4.5%) (P=0.710). Patients with dryness of mouth and hoarse voice were comparable in both groups. Patient-reported loss of taste was comparable in both arms (P=0.731) (Table 3).

**Table 3:** Comparison of patient-reported symptoms.

Toxicity	Study (%)	Control (%)	Total (%)	P-value
Pain throat				
Not at all	1(4.3)	1(4.5)	2(4.4)	0.710
A little	9(39.1)	10(45.4)	19(42.2)	
Quite a bit	11(47.8)	11(50)	22(48.8)	
Very much	2(8.6)	0	2(4.4)	
Dry mouth				
Not at all	0	2(9)	2(4.4)	0.186
A little	5(21.7)	8(36.3)	13(28.8)	
Quite a bit	15(65.2)	11(50)	26(57.7)	
Very much	2(8.6)	1(4.5)	3(6.6)6	
Post-treatment hoarseness of voice				
Not at all	2(8.6)	0	2(4.4)	0.939
A little	8(34.7)	7(31.8)	15(33.3)	
Quite a bit	11(47.8)	14(63.6)	25(55.5)	
Very much	2(8.6)	1(4.5)	3(6.66)	
Loss of taste				
Not at all	2(8.6)	0	2(4.4)	0.731
A little	5(21.7)	4(18.1)	9(20)	
Quite a bit	12(52)	14(63.6)	26(57.7)	
Very much	4(17.3)	3(18.1)	7(15.5)	

## Discussion

In this study, the toxicities of concomitant boost radiotherapy and concurrent chemoradiation were compared. The mean time to treatment completion was 37 days in the study arm (CBRT) and 49 days in the control (CRT) arm. Interruption in treatment due to acute toxicities was seen in 4(17.4%) patients in the study group and in 6(27.3%) patients in the control group, however, the difference was not statistically significant. The overall response rates for the primary and nodal disease at first follow-up were comparable, with insignificant p-values (p=0.468 and p=0.218).

Grades II and III local toxicities were higher in the concomitant boost arm, and the difference was statistically significant for grade III laryngeal toxicity (P=0.029). Haematological and renal toxicities were higher in the CRT arm which was statistically significant (P-0004 and 0.018, respectively).

Delayed confluent mucositis was seen in three patients in the CBRT arm and in one patient in the CRT arm, the difference was statistically insignificant. Delayed skin reactions were also comparable in both arms. All delayed toxicities were transient and healed after eight weeks of treatment completion.

Patients' quality of life was also compared between the two arms by documenting patient-reported symptoms of pain in the throat, dry mouth, hoarseness of voice, and loss of taste, which were found to be comparable in both groups.

In a previous study by Ghoshal et al., the grade III/IV mucosal and dermal toxicities in the concomitant boost radiotherapy arm (35%) were significantly higher than those in the conventional radiotherapy arm (19%). These are comparable to 39% in the CBRT arm and 22% in the CRT arm in our study. The slight increase in toxicity in both arms in our study could be because all patients were treated conventionally with Cobalt 60, whereas in the study by Ghoshal et al., 6 MV photons were also used for treatment delivery. Also, we gave concurrent chemotherapy in the conventional radiotherapy arm, which increased toxicity in the control arm.

In the RTOG 9003 trial, which compared the different radiotherapy fractionation schedules, the acute and late grade III or higher toxicities were 58.8% and 37.2% for accelerated fractionation with concomitant boost, respectively. Both were significantly higher as compared to the conventional fractionation arm P=0.0001 and P=0.011, respectively. Compared to our study, higher toxicity in the CBRT arm in RTOG 9003 could be because of the higher overall radiotherapy dose given, i.e., 72 Gy/42 fractions/6 weeks compared to 67.5 Gy/40#/5 weeks used in this study.

In two retrospective studies by Narvaez et al., a non-significant difference in toxicity was observed between concomitant boost radiotherapy and conventional chemoradiotherapy; however, these studies were limited by a small sample size [29,30].

In this study, overall local toxicities and grade IV toxicities were higher in the concomitant boost arm but did not lead to prolonged periods of treatment interruption, and treatment was completed significantly earlier as compared to CRT.

Patient-reported symptoms pertaining to quality of life were also found to be comparable with CRT. With the advent of modern radiotherapy, the acute toxicities associated with accelerated fractionation regimens can be further reduced. As per this trial, concomitant boost radiotherapy was found to be a safe and feasible option in locally advanced head and neck cancer patients, with comparable toxicity to concurrent chemoradiation.

The limitations of this study were the small sample size, which can be responsible for the insignificant difference in toxicities. Due to a short follow-up, late toxicities were not assessed. For the assessment of patient-reported quality of life, a standardized questionnaire would have been a more reliable method.

## Conclusions

Accelerated radiotherapy with a concomitant boost can be considered a potential alternate treatment option for patients who are not candidates for concurrent chemoradiation. The overall local toxicities to the site of radiation appear similar between the two arms, except for higher grade III laryngeal toxicity in the concomitant boost arm. With modern radiotherapy techniques, local treatment toxicity can be further reduced. Concomitant boost radiotherapy is associated with decreased risk for hematologic toxicities. In addition, CBRT carries the appealing advantage of early treatment completion and a reduced patient waiting list in busy radiotherapy departments.

However, this was a small study, and larger prospective randomized studies are needed to evaluate the role of concomitant boost radiotherapy further.
